# *“I passed the test!”* Evidence of diagnostic misconception in the recruitment of population controls for an H3Africa genomic study in Cape Town, South Africa

**DOI:** 10.1186/s12910-017-0175-z

**Published:** 2017-02-15

**Authors:** Francis Masiye, Bongani Mayosi, Jantina de Vries

**Affiliations:** 1 Department of Medicine, University of Cape Town, Faculty of Health Sciences, Cape Town, South Africa; 2 University of Malawi College of Medicine, School of Public Health, Centre for Bioethics in Eastern and Southern Africa (CEBESA), Private Bag 360, Chichiri, Blantyre, Malawi

**Keywords:** Diagnostic misconception, Therapeutic misconception, Genetic studies, Genomic studies, In-depth interviews, Informed consent, Observations, Qualitative study

## Abstract

**Background:**

Advances in genetic and genomic research have introduced challenges in obtaining informed consent for research in low and middle-income settings. However, there are only few studies that have explored challenges in obtaining informed consent in genetic and genomic research in Africa and none in South Africa. To start filling this gap, we conducted an empirical study to investigate the efficacy of informed consent procedures for an H3Africa genomic study on Rheumatic Heart Disease (RHDGen) at the University of Cape Town in South Africa. The main aim of the study was to understand ethical challenges in obtaining informed consent in the RHDGen study.

**Methods:**

We used a qualitative study methodology involving in-depth interviews and participant observations. Our study participants were RHDGen cases (patients), healthy controls and research staff involved in the recruitment of RHDGen cases and controls. In total, we conducted 32 in-depth interviews with RHDGen cases and controls, 2 in-depth interviews with research staff and 57 direct observations of the consent procedures of RHDGen cases and controls. The interviews were conducted in English, audio-recorded and transcribed verbatim. Data were analyzed using thematic content analysis. The study was conducted in 3 sites within Cape Town, South Africa.

**Results:**

Most healthy controls joined the RHDGen study in order to be screened for rheumatic heart disease (diagnostic misconception). A majority of RHDGen cases decided to join the RHDGen study because of therapeutic misconception.

**Conclusion:**

The ethical challenges that impacted on obtaining informed consent in the RHDGen study were complex. In this study, the main challenges were diagnostic misconception among RHDGen controls and therapeutic misconception among RHDGen cases.

## Background

Informed consent is paramount for the ethical conduct of research [1–11]. However, empirical research shows that obtaining genuine informed consent in biomedical research is difficult in practice in both high and resource limited settings [1]. In addition, advances in genetic and genomic research have introduced new challenges in obtaining informed consent in research practice [12]. Some of the new ethical challenges include granting broad consent for use of samples and data in future research [13–17]; the difficulty in explaining to potential research participants the sharing of data and samples with researchers who were not part of the original study in which samples and data were collected; the challenge of returning incidental or unrelated findings that were not part of the original research project to research participants [18–22]; and the difficulty in explaining risks not only to individual genomic research participants but also to their families, communities and even ethnic groups in cases where members share the same genetic mutation associated with increased risk of stigmatization [15–22].

So far, there are few studies that have explored challenges in obtaining informed consent for genetic and genomic research in Africa [12–19]. Specific ethical challenges in informed consent that have been previously identified in African genomic research include the difficulty in explaining scientific methods and concepts such as “gene”, “genetics”, “genomics”, “DNA”, “genetic database” and “data release” in local languages during the consent process; the conduct of research in emergency situations which make standard consent processes impracticable especially where patients or their guardians are under stress; therapeutic misconception among research participants who are recruited in clinical settings and who have the widespread conception that research studies result in clinical benefit; the trust that research participants have in medical doctors who are also researchers [15–19]; and the possible risk of stigma or exploitation of study communities. There is also a regulatory gap and limited legal and ethical guidance available in Africa to support a transition from specific consent to broad consent models [23]. These ethical challenges are frequently compounded by low literacy, poverty, socio-cultural barriers and ineffective regulatory mechanisms in low and middle income countries.

In addition, none of the published papers specifically consider the implications of recruiting healthy controls for genomic research in Africa generally and in South Africa specifically. Interestingly, there is virtually no work that has been done in South Africa on ethical challenges in obtaining informed consent for genomic research in general and the implications of recruiting healthy controls for genomic research in particular. To start filling this gap, we conducted an empirical study to investigate the efficacy of the informed consent procedures for a genomic study on Rheumatic Heart Disease that is currently being conducted in the Department of Medicine at the University of Cape Town in South Africa. The main aim of the study was to understand ethical challenges in obtaining informed consent in the RHDGen study.

### Background of the genomics of rheumatic heart disease network (RHDGen) study

The RHDGen Study is one of the genomic research projects funded by the Wellcome Trust under the H3Africa Consortium. Rheumatic Heart Disease (RHD) is a chronic heart condition that is caused by Rheumatic Fever (RF). RF is caused by a bacterium called *Streptococcus pyogenes* that make some people have a sore throat. While some people get better from this infection, others develop rheumatic heart disease. However, the infection can be prevented from developing into rheumatic heart disease by taking antibiotics such as penicillin [24–26]. Currently, RHD remains the most common cardiovascular disease in young people under the age of 25 worldwide and it manifests itself with heart failure, stroke, infective endocarditis and pregnancy-related complications [27]. Whilst scientists do not understand why some people with the infection develop RHD while others do not develop the disease, it is likely that genetics is a contributing factor [28]. The RHDGen study is recruiting 2500 adult patients (cases) with echocardiographically-confirmed RHD and it will compare their data with that of 3500 non-affected individuals (healthy population controls) from 8 sub-Saharan African countries in order to identify genetic factors of risk. The eight countries involved in the RHDGen network are Kenya, Mozambique, Namibia, Nigeria, South Africa, Sudan, Uganda and Zambia. Written informed consent is obtained from both prospective RHDGen cases and controls before their participation in the study. RHDGen controls who are found to have cardiac problems during the screening procedures into the RHDGen research project are referred to Cardiac Clinics for medical attention. This study on ethical challenges in obtaining informed consent in the RHDGen study was nested in the RHDGen study in South Africa specifically in the Western Cape where both RHD cases and controls were being recruited. For clarity in this paper, participants recruited for the RHDGen study will be referred to as ‘cases and controls recruited in the RHDGen study’. Participants recruited for the qualitative study described in this paper will be referred to as ‘participants for the research study’, or “participants”.

## Methods

### Study design

The study used a cross-sectional study design to collect data from research participants. This design was appropriate for the study because data was collected from research participants at one point in time thereby allowing the acquisition of data in an open, flexible and inductive manner. Since the main research question was to understand ethical challenges in obtaining informed consent in genomic research, the study was exploratory and descriptive in nature.

### Data collection methods, sampling and recruitment of study participants

A combination of two qualitative research methods – in-depth interviews (IDIs) and participant observations (POs) – were employed in the study. The qualitative study approach employed in this study allowed the researchers to derive in-depth information from study participants.

Before conducting each IDI, individual written informed consent was obtained from each of the respondents and a demographic data form was administered to document respondents’ age, sex, highest education achieved, occupation, religion, first language, the location where the respondent was living, the type of respondent and the time since the study participant was recruited into the RHDGen research project. These demographic data were used in the analysis process of the interviews to compare responses from different study participants based on their demographic information Semi-structured interview guides were used to guide the interviews with cases and controls recruited into the RHDGen research project and research staff in order to get in-depth knowledge and experience on the consent process. The interview guides had sets of open-ended questions and the questions were formulated using the study objectives and they were being revised as the data collection progressed.

The IDIs with cases in the Cardiac Clinic of the Groote Schuur Hospital and with controls at the Vanguard Community Health Centre and the Heideveld Community as well as with research staff were conducted by one researcher (FM). The IDIs were conducted in English and consent was obtained for audio recording. Audio-recordings were transcribed verbatim. Notes were taken and observations were recorded at each interview. All interviews were conducted on the day of enrolment in the RHDGen study.

Both male and female cases and controls recruited into the RHDGen research project were included in this study. For the RHDGen cases, the IDIs were conducted in the recruitment room located in the Cardiac Clinic of the Groote Schuur Hospital. Twelve (12) IDIs were conducted there with RHDGen cases. For RHDGen controls, IDIs were conducted either in the front seat of the van which was used for recruitment at the Vanguard Community Health Centre and the Heideveld Community or in the meeting room of the Clinical Research Centre in the Old Main Building of the Groote Schuur Hospital. Twenty (20) IDIs were conducted with RHDGen controls at the Vanguard Community Health Centre (9), Heideveld Community (5) and the Clinical Research Centre in the Old Main Building of the Groote Schuur Hospital (6). In total, 34 IDIs were conducted with RHDGen cases and controls.

The study also targeted research nurses who were responsible for consenting potential RHDGen cases and controls. The research nurses were scheduled for IDIs at the time of their convenience. Two (2) IDIs were conducted with research nurses involved in the consenting and recruitment of RHDGen cases and controls. Both IDIs with research nurses were conducted by the researcher.

In addition to in-depth interviews, we also conducted a large number of participant observations for this project. All POs were conducted by one of us. For the POs, the research nurse administering the consent process introduced the researcher orally before commencing the RHDGen consent process. The researcher then sought verbal consent from each potential study participant for his presence during the consenting process. All study participants in the POs were given an information leaflet to inform them about the study. During the observations, the researcher took notes which were typed up immediately after the observations. The information obtained in the POs informed the topic guides for IDIs with both RHDGen cases and controls as well as research staff. Some of the POs were succeeded by IDIs with both RHDGen cases and controls.

The POs with RHDGen cases and controls were conducted at the same venues where they were recruited into the RHDGen research project while the IDIs with research staff were conducted at the Clinical Research Centre in the Old Main Building of the Groote Schuur Hospital.

Data were collected from RHDGen cases and controls as well as research staff for a period of 5 months from July to November 2014.

In total, 91 study participants were recruited into the study. Thirty-four (34) participants took part in the in-depth interviews while 57 participants took part in the observations.

### Study setting

As highlighted above, participants for this study were recruited in the Cardiac Clinic and the Clinical Research Centre of the Groote Schuur Hospital as well as the Vanguard Community Health Centre and the Heideveld Community within Cape Town in South Africa. The study enrolled RHDGen cases and controls as well RHDGen research staff involved in recruiting RHDGen cases and controls. The RHDGen cases were recruited from the Cardiac Clinic at the Groote Schuur Hospital. Controls for the RHDGen research project were recruited at the Vanguard Community Health Centre in Bonteheuwel, the Heideveld Community in the Cape Flats and the Clinical Research Centre of the Groote Schuur Hospital. All the controls for the RHDGen research project were people who did not suffer from any chronic diseases, including heart disease, at the time of recruitment.

The study conducted observations of the consent process and in-depth interviews with RHDGen cases and controls across all these RHDGen recruitment sites. The RHDGen staff were recruited and interviewed at the Clinical Research Centre.

Participant observations of the consent process of both RHDGen cases and controls were conducted in the Cardiac Clinic and Clinical Research Centre at the Groote Schuur Hospital, the Vanguard Community Health Centre and the Heideveld Community.

### Data processing and management

All interviews were conducted in English and recordings were transcribed verbatim. Each transcript had a preamble or summary of the interview which was informed by the notes that were taken during the interviews. All transcripts were imported into NVivo 10 for analysis.

Notes were taken during the POs and typed up immediately after each PO. The written notes from the POs were read thoroughly in order to understand the dynamics in the recruitment and consenting process of the RHDGen cases and controls.

### Data analysis

Thematic content analysis was used to analyze interview data. The analysis was iterative and preliminary analysis started immediately when the initial interviews were transcribed. Two researchers were involved in the analysis and we used a progressive coding strategy where we first assigned open codes to interesting or relevant text excerpts. These codes were descriptive of the content of the quotes. Once we had gone through a small number of interviews and did not find any additional codes, we looked at the list of open codes and started to develop a hierarchical coding scheme, identifying what appeared as overarching themes and codes underneath those. After piloting and adapting that initial coding scheme, we developed a final hierarchical coding scheme that was applied to all the interviews in our dataset. The higher hierarchical codes were grouped into main themes with the lower nodes as their sub-themes. The transcripts were re-coded using this new hierarchical coding scheme. Memos were written for each hierarchical code. We reviewed and discussed the quotes for each theme. Data summaries were produced from the texts. The data summaries comprised main themes and their sub-themes as well as their quotes as examples from the texts. We discussed the data summaries and from the discussions, a charting framework was developed with linkages to the data. The charts were produced for each main theme and sub-theme. The charts were also compared across all themes. Each chart was given a descriptive account of each theme and had relevant quotes from the study participants. The charts were used to develop a first account of the empirical data, which we discussed. Subsequent drafts re-examined the data and the charts, and integrated emerging insights into the analysis.

### Ethical considerations

Ethics approval for this study was obtained from the Human Subjects Research Review Committee in the Faculty of Health Sciences at the University of Cape Town (FHS HREC 251–2014). Written consent was obtained from each of the study participants prior to their participation in the IDIs while verbal consent was obtained from all the participants who participated in the participant observations. Verbal consent was obtained from the potential study participants to have the consent process observed because there was no interaction with the RHDGen cases and controls during the consent process other than observing the consent process. Individual written informed consent was sought from each of the research nurses administering the consent procedures once before the first participant observation was conducted.

## Results

### Demographic characteristics of study participants

A total of 34 IDIs and 57 POs were conducted with RHD cases and controls as well as RHDGen research staff. Most study participants were females and aged between 18 and 40. Table 1 below gives demographic characteristics of the study participants.

**Table 1:** Demographic Characteristics of Study Participants

Demographic Characteristics		IDI-Respondents (*N* = 34)	Of which cases (*N* = 12)	Of which controls (*N* = 20)	Of which Research Staff (*N* = 2)	Percentages (100%)
Gender	Male	10	4	6	0	29%
	Female	24	8	14	2	71%
Age	18-40	21	9	11	1	62%
	41-61	13	3	9	1	38%
First language	Xhosa	17	9	8	0	50%
	Afrikaans	14	3	10	1	41%
	English	3	0	2	1	9%
Occupation	Employed	23	10	11	2	68%
	Not employed	11	2	9	0	32%
Religion	Christian	26	6	14	2	76%
	Muslim	6	3	4	0	18%
	Non-religious	2	3	2	0	6%
Highest level of education achieved	Primary	2	0	2	0	6%
	Secondary	24	8	16	0	71%
	Tertiary	8	4	2	2	23%

### Diagnostic misconception as a motivation for study participation among RHDGen controls

In this paper, we focus our analysis on the factors that motivated healthy people to participate in the genomics research project we were concerned with. These participants described a variety of reasons to participate in the study, including for instance altruism, trust and familiarity with research staff. However, the most common motivation for study participation among the RHDGen controls was diagnostic misconception.

### Unmet health care needs as a contribution to diagnostic misconception among the RHDGen controls

One of the most often expressed motivators related to curiosity about ‘seeing one’s heart’ beat on a monitor, and finding out whether one’s heart was healthy.

*The thing that motivated me was to know if I have a heart disease and I wanted to see my heart beating too* (IDI # COV 02).

*I was interested to know if I have a heart problem and I am happy that I don’t have any heart problem* (IDI # COV 04).

*Yes, it is helpful because people are able to know whether they have a heart problem. For me I was very happy when the doctor told me that I don’t have any heart problem* (IDI with study participant # COC 21).

The consent process for this genomic study included information about Rheumatic Heart Disease, which is the disease that was studied in the genomic study. But whilst it makes sense for the consent process for RHD patients (recruited as ‘cases’) to be tied to the illness that the genomic study focuses on, this focus appeared confusing for control participants who were not ill with heart disease.

What participants seemed to retain most strongly about the study is that they were informed that they would have heart scans and the doctor would be able to tell them if they had any heart problems or conditions.

*I just wanted to check my heart if I had a problem or not* (IDI # COV 01).

*The main reason why people decided to come is because of the screening for heart problems. We were told you had those machines for screening people and since most of us do not know whether we have heart problems or not, it was necessary to come for the screening* (IDI # COH 30).

During participant observations, we found that many participants approached research participation a bit like they may approach an examination – with initial apprehension, and then full of joy and happiness after going through the screening program and after being told that they did not have rheumatic heart disease. Some came out of the bus with their arms in the air, saying “I passed the test!”

Many of the controls we interviewed referred to the RHDGen study as a screening study, offering a free service that is rarely available in their communities. For instance,

*I think the other reason is that this is a free service and such free services are rarely found here. So, I thought it was necessary to have the service* (IDI # COV 04).

*We don’t have check-ups for heart problems here and sometimes they don’t test our blood when we are sick. So, with these free check-ups, I decided to come* (IDI # COV 10).

The impression that the RHDGen study involved a screening programme was reinforced in two important ways: first, because recruitment took place in a van that was equipped with ECG and ECHO machines, and that carried colourful branding of the university, the research group and the disease we work on. Study participants were familiar with other screening activities that take place in these communities from time to time – for breast cancer, HIV and TB for instance. Such screening endeavours employ a similar van, branded in a similar way, and so the assumption that this genomic research project was also a screening project seems logical. Thus,

*I asked the sister to test me for TB and HIV too because my brother was tested for TB by the same people when they came to test people for TB and HIV in our community last year. But the sister told me that they are not testing people for TB and HIV this time. Instead they are testing people for heart disease. So, they tested me for the heart disease but the machine did not find it.* (IDI # COV 13).

*Well I work for an organization that runs clinical trials. So, it was quite interesting to be the participant and decided to consent and to be informed about this study. I came partly because I was quite keen to have an ECHO and an ECG; for me, it was a way of testing my heart to see if it was healthy …* (IDI # COC 20).

### Community leader contributions to diagnostic misconception among RHDGen controls

The impression that the RHDGen study involved screening was reinforced by the research groups’ reliance on community leaders to identify research participants. Particularly in one of the four recruitment sites, the research team had built relationships with a community leader who assured the team of safety and hosted the team when recruiting – for instance, by making his home available to the research nurse for blood collection. This community leader seemed eager to please and ensure that the group reached target recruitment numbers. In ensuring that enough people showed up on the right date, he had apparently informed community members that there was going to be a free screening program on rheumatic heart disease by doctors from Groote Schuur Hospital.

*I was asked to come by our chairman. He announced two days ago that doctors from the Groote Schuur Hospital will come today and every Monday to screen people for rheumatic heart disease. So, that is why I came today* (IDI # COH 26).

*The community leader announced to everybody that you would be coming to this community to check people for rheumatic heart disease* (IDI # COH 32).

An interesting contributory factor lending further support to the importance of the diagnostic misconception in the RHDGen enrolment is that many controls also described that they joined the RHDGen study because of stories they had heard of people who had heart complications and died because of ignorance about the status of their heart conditions, for instance,

*Because another thing that made me get interested, my sister in law died of a heart disease. She had a heart problem and so that’s what interested me to know if I have a heart problem. I was also scared of passing away from such a disease* (IDI # COV 02).

*The doctor put something on my chest and I could see my heart moving and he recorded everything on that machine. After that, I went to the third room where I could see the beating of my heart and the lady there told me that I don’t have any heart problem and my heart condition is fine. Obviously, I was happy to hear that because I was worried about my heart since I feel pain at the chest at times* (IDI # COH 30).

### Logistical contributions to diagnostic misconception among RHDGen controls

One of the RHDGen research staff who participated in the in-depth interviews noted that lack of understanding due to language barriers might have contributed to the misconception that the study was a screening program for heart conditions. She also observed that some of the RHDGen controls had already made up their mind to have the screening before they went through the consent process and that it was difficult for such controls to understand that this was a research activity and not a screening program. Thus she said:

*Ok I think the first challenge is probably the language barrier because I don’t speak Xhosa. And so, that’s a big challenge to some patients, although they might speak English but their English isn’t good. Another challenge is some patients come when they have already made up their mind to be screened for heart conditions. So, even when you give them information that this is research, they seem not to understand* (IDI # RS 02).

### Evidence of therapeutic misconception as a motivation for study participation among RHDGen cases

By contrast, when we examined the data from interviews with RHD patients recruited as cases, we did not find such strong evidence of a diagnostic misconception. However, RHD patients linked their motivations to participate more clearly to the condition under study, and to the hope that this study could help in the identification of new therapies for RHD. For instance, the RHDGen cases described that the current drugs that are being given to patients with rheumatic heart disease such as warfarin can have devastating side effects and they felt that through the RHDGen study, doctors and scientists would be able to develop better drugs for the current RHD patients and other patients in the future.

*I think it will also help doctors to come up with new and better medications for this disease because they will understand what happens in the body when one has the disease by studying the DNA. In such a way, patients with rheumatic heart disease will be treated better and other patients with this disease will not have complications in the future* (IDI # CWR 18).

*Aah I think the issue of finding better drugs for people who suffer from this disease is important* (IDI # CWR 15).

*As I said they may also develop better medicines to treat the disease* (IDI # CWR 18).

*I joined because this study is useful. As I said it will help in developing better treatment and prevention for people with the sore throat, fever and those with the disease* (IDI # CWR 15).

While some RHDGen cases recruited in the Cardiac Clinic at the Groote Schuur Hospital reported an expectation that the drugs that would be developed from this study could help them in improving their own health, other patients anticipated that they would derive direct personal benefit in the form of better treatment from the drugs that would be developed from the results of the RHDGen study. The expectation to receive better treatment because of their participation in the RHDGen study has been described as ‘therapeutic misconception’ in the ethics literature. Therapeutic misconception depicts the hope that research participants may experience when previous clinical treatments have failed to help them adequately and hope to obtain better treatment by participating in research [28].

## Discussion

From the findings of this study, it is evident that the majority of RHDGen controls chose to participate in the RHDGen study because they were motivated by knowledge about heart disease, heart conditions, blood pressure, general health status and other medical conditions. This specific challenge has been termed ‘diagnostic misconception” in literature describing challenges in the consent process for genomics research. It is a unique challenge that arises in the context of genomics research and similar studies that recruit thousands of healthy participants in a study that is usually disease-specific, using consent forms that are designed around the disease in question. The diagnostic misconception arises when research participants do not appreciate the difference between research and diagnosis [20]. It has been identified as an equivalent of therapeutic misconception in the genetic research context [20]. According to literature, diagnostic misconception occurs when potential research participants consider research participation as an opportunity to be checked or diagnosed for diseases [22, 29]. In some cases, participants may believe that they are being enrolled in research in order to be checked for the disease under study while in other cases, research participants may believe that they are being enrolled in research in order to receive individualized information about medical diagnoses and future disease risks [20, 21, 30]. This is contrasted with therapeutic misconception that occurs in clinical trials when research participants either misunderstand or fail to appreciate the key differences between research and clinical care [31–34]. Currently, there is not much empirical data on diagnostic misconception in genetic studies and none in genomic studies in Africa. Therefore, this paper constitutes the first evidence of diagnostic misconception as an ethical challenge to genomic studies in Africa.

One important reason that the diagnostic misconception arose strongly in our study relates to the relatively resource-limited backgrounds of many of our study participants. Rheumatic Heart Disease is a poverty-related condition that is more prevalent in poorer communities with crowded housing [35]. Because of a scientific need to closely match cases and controls in terms of ethnicity and age, the research team recruited controls from communities attending the Vanguard Community Health Centre and living in Heideveld. Both of these are resource-limited communities with low average income, limited access to healthcare services, and high rates of crime. In these communities, the opportunity to benefit from what was perceived to be a free healthcare service is significant and important to study participants.

What also contributed to the diagnostic misconception is that recruitment took place in a van that prominently displayed public health messages relating to Rheumatic Heart Disease and children’s health - a remnant of a previous research project by the group. This van is similar to those used by other public health screening endeavours, including for instance breast cancer, TB and HIV screening. This similarity caused expectations that participation in the project constituted undergoing a medical examination for heart disease, rather than mere participation in a research project. The recruitment of large numbers of healthy participants, who had not previously heard of rheumatic heart disease, further increased the incidence of diagnostic misconception.

## Conclusion

Based on the information which was obtained from participant observations during the recruitment process of both RHD cases and controls as well as in-depth interviews with RHDGen cases, controls and research staff involved in obtaining informed consent from research participants, most RHDGen controls had beliefs related to diagnostic misconception while RHDGen cases were motivated by therapeutic misconception. The RHDGen controls mistakenly considered the RHDGen study as a free screening program for heart conditions while RHDGen cases considered it as a drug trial for rheumatic heart disease. In order to avoid diagnostic misconception and therapeutic misconception in future genomic studies, there is need for provision of adequate information about research objectives and procedures including anticipated benefits and risks to potential research participants before obtaining their consent to participate in genomic studies. There is also need to assess potential research participants’ understanding or comprehension about scientific terms and concepts used in genomic research prior to enrolling them in genomic studies. In addition, research staff who are involved in recruiting potential research participants for genomic studies must be creative during the recruitment process and ensure that the studies are conducted properly without compromising ethics. Consent processes must be flexible enough to allow staff to adjust these to the specific recruitment context and ensure that potential research participants make informed decisions. Moreover, the design of consent forms and processes for genomic research should not be based on checklists for consent documents but they must be informed by experiences of researchers and field staff working in communities where the intended research projects are to be conducted. For instance, the consent process for recruiting potential research participants from communities that are unsafe and insecure for research staff must be informed by inputs from community leaders and researchers who have experience in conducting research in such communities. Furthermore, there is need to improve communication with and education of community partners who are involved in identifying potential research participants for recruitment into research studies. Research staff must involve both community partners and potential research participants in sensitization activities where they have to explain scientific terms and concepts used in genomic research and allow them to ask questions before approaching individuals to take part in their genomic research projects. Such community education should ideally involve all community members. In communities where community advisory boards (CABs) exist, CAB members should be involved in communicating scientific terms and concepts in lay terms to their fellow community members. In communities where CABs do not exist, it is important to establish CABs to assist research staff in sensitization activities and communicating information about genomics, DNA and data sharing prior to approaching individual potential research participants to join genomic studies. Finally, it is necessary to train research staff responsible for recruiting research participants in the protocol and the informed consent process before initiating genomic research projects. After such protocol and informed consent process training, research staff should conduct pre-testing (piloting) of data collection tools and informed consent documents among lay community members and make appropriate corrections or revisions to the data collection tools and informed consent documents before implementing genomic studies.

## Abbreviations

AIDS: Acquired immuno-deficiency syndrome
DNA: Deoxyribo-nucleic acid
ECG: Electrocardiogram
ECHO: Echocardiogram
H3A: Human health and heredity in Africa
HIV: Human Immunodeficiency virus
IDI: In-depth interviews
PO: Participant observation
RHD: Rheumatic heart disease
RHDGen: Genomics of rheumatic heart disease
TB: Tuberculosis
UCT: University of Cape Town
